# Bigeminal potentials in the pulmonary vein indicate arrhythmogenic trigger of atrial fibrillation

**DOI:** 10.1002/joa3.12462

**Published:** 2021-01-19

**Authors:** Yasushi Mukai, Shunsuke Kawai, Shujiro Inoue, Susumu Takase, Kazuo Sakamoto, Kazuhiro Nagaoka, Akiko Chishaki, Hiroyuki Tsutsui

**Affiliations:** ^1^ Division of Cardiology Japanese Red‐Dross Fukuoka Hospital Fukuoka Japan; ^2^ Department of Cardiovascular Medicine Kyushu University Hospital Fukuoka Japan; ^3^ Division of Cardiology Aso Iizuka Hospital Iizuka Japan; ^4^ Division of Cardiology St. Mary’s Hospital Kurume Japan; ^5^ Division of Cardiology Fukuoka Dental College Hospital Fukuoka Japan

**Keywords:** arrhythmogenic trigger, atrial fibrillation, pulmonary vein, PV bigeminy

## Abstract

**Background:**

The pulmonary veins (PVs) have unique electrophysiological properties triggering and maintaining atrial fibrillation (AF). Bigeminal PV electrical activity (PV bigeminy) during sinus rhythm has been reported; however, its mechanisms and clinical implication remain unclear. We hypothesized that PV bigeminy indicates arrhythmogenic activities and influences clinical outcome.

**Methods and Results:**

We retrospectively analyzed electrophysiological studies in 465 patients with AF who underwent first session PV isolation (PVI). PV bigeminy was observed in 30 PVs of 23 patients (4.9% of patients). PV bigeminy was observed in left inferior PV (LIPV) in 15 patients, which was the most prevalent, followed by left superior in seven and right superior in seven and right inferior in one. In response to atrial extra stimulus, the second PV potentials (PV2) showed decremental conduction properties, suggesting reentrant mechanisms involved (n = 5). Interestingly, AF was initiated from the 23 PVs with bigeminy in 21 patients (76.7% of 30 PVs with bigeminy), spontaneously or in response to drugs, which was significantly more prevalent from the AF initiation rate from each PV in the control 442 patients (182 firings in 1290 PVs, 14.1%, *P* < .0001). PVI‐based ablation was completed in the 23 patients with PV bigeminy and no recurrence was observed during 1‐year follow‐up, whereas four patients needed second sessions.

**Conclusions:**

PV bigeminy is relatively rare but a unique electrophysiological finding in AF patients, suggesting reentrant substrate within the PV and/or surrounding tissue. PV bigeminy is a strong indicator of arrhythmogenic vein triggering AF, and ensures an excellent clinical outcome after PVI.

## Graphical abstract

PV bigeminy is relatively rare but a unique electrophysiological finding that indicates arrhythmogenic PV triggering AF.

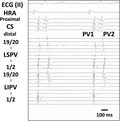

## 1 INTRODUCTION

Pulmonary veins (PVs) are the major arrhythmogenic trigger sites of atrial fibrillation (AF).^1^, ^2^ Ectopic activities and complex conduction properties are the crucial electrical characteristics of PVs, which initiate and maintain AF.^3^, ^4^ Variety of unique electrical activities in PV have been reported.^5^ Bigeminal PV potentials (PV bigeminy) are rare phenomenon characterized by a second series of PV potential that is separate in time phase from the ordinary PV potentials observed in the PV ostia during sinus rhythm. Pathophysiology and clinical implications of PV bigeminy still remain unclear.^6^, ^7^, ^8^, ^9^, ^10^, ^11^ A recent study has suggested that a concealed PV bigeminy may be an indicator of arrhythmogenic activity.^11^ In this study, we aimed to characterize PV bigeminy and clarify their roles in AF.

## 2 METHODS

### 2.1 Study subjects

We retrospectively analyzed 465 patients who underwent first session catheter ablation for symptomatic AF from 2011 to 2018. Paroxysmal AF (pAF) and non‐paroxysmal AF (non‐pAF) were defined according to the guideline of the American College of Cardiology/American Heart Association/Heart Rhythm Society.^12^ All patients underwent therapeutic protocols based on standard extensive PV isolation (PVI). Antiarrhythmic drugs (AADs) were discontinued for at least five half‐lives prior to ablation procedures. This study was in compliance with the principles outlined in the Declaration of Helsinki and was approved by the institutional review board for ethics at our institution, Kyushu University Hospital (approval no. 29‐44). An informed consent was obtained in the form of opt‐out on the website: https://www.cardiol.med.kyushu‐u.ac.jp/research/clinical‐research/.

### 2.2 Electrophysiological study

A duodeca‐polar electrode catheter was inserted to the coronary sinus (CS) from jugular vein. We accessed the left atrium (LA) through transseptal catheterization with three sheaths. A left arteriography was performed to display anatomical information of the LA and four PVs. We applied two circular, duodeca‐polar electrode catheters for circumferential PV mapping to both upper and lower PVs. The other route was used for an ablation catheter.^2^ PV mapping was performed with a steerable circular catheter of 15 to 25 mm in diameter according to each PV size. Catheter size was selected based on the predetermined measurement of the PV diameter on venography or computed tomography. Prior to ablation procedure, electrograms were recorded in all four PVs. In left PVs, control record was also obtained under atrial pacing from distal site of CS. If bigeminal electrical activities of PV were consistently observed, a series of overdrive atrial pacing with extra stimuli was attempted to observe responses of these potentials. PV bigeminy was defined as an existence of consistent second PV potential(s), which is separated in time phase by the first PV potentials during sinus rhythm or atrial pacing.

### 2.3 AF induction test

**FIGURE 1:**
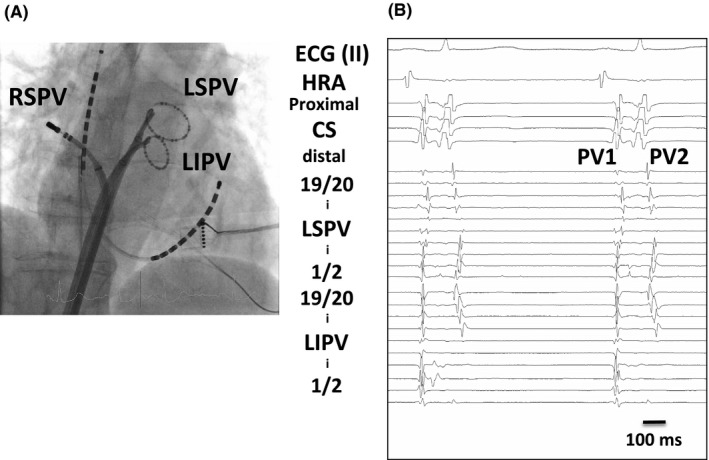
A, An electrode position during electrophysiological study and AF induction test. B, Bigeminal PV potentials (PV bigeminy: PV1 and PV2) during sinus rhythm

Isoproterenol (5 μg) was administered intravenously to induce AF or PV firing. If it remained on sinus rhythm, adenosine triphosphate (ATP, 20 mg) was further administered. If AF or PV firing was induced, earliest activation site was defined as an AF trigger site. Three PVs (left superior, left inferior, and right superior) were monitored simultaneously using two multipolar electrodes and an ablation catheter unless spontaneous arrhythmogenic activity of right inferior PV was observed^2^ (Figure 1A).

### 2.4 AF ablation procedure

We performed PVI‐based ablation in all patients. A 3.5 mm open‐irrigated‐tip ablation catheter was used (Navistar Thermocool, Biosense Webster). Radiofrequency energy was delivered with a maximum temperature setting of 43°C and a power of 20‐35 W. We defined successful PVI as the loss of PV potentials during sinus rhythm or CS pacing (entrance block) and local PV capture without atrial capture by pacing from a circular mapping catheter or an ablation catheter placed in the PVs just distal to the radiofrequency ablation lesions (exit block).^13^ Linear ablation (LA roof line/mitral isthmus line) or defragmentation ablations were added in some patients at attending operators’ decisions.

### 2.5 Follow‐up

No AADs were prescribed after 3 months of a blanking period of index procedures for 23 patients. Patients underwent continuous in‐hospital electrocardiographic monitoring for 2 days after the procedure. Patients underwent observation of every month at the outpatient clinic. The outcome of AF ablation was evaluated on the basis of patient's symptoms and periodic 24 hour electrocardiogram at 3, 6, and 12 months after the procedure. AF recurrence was defined as AF lasting for >60 seconds after a blanking period of 3 months.

### 2.6 Statistical analysis

The data are expressed as mean ± standard deviation (SD) for continuous variables, and counts and percentages for categorical variables. A comparison of categorical variables between pairs of groups was carried out using the Chi‐square test or Fisher's exact test. A comparison of continuous variables between pairs of groups was carried out using Student's *t*‐test. All *P*‐values <.05 were considered significant. Analyses were conducted using a software program JMP (SAS, Cary, NC). The power for the log‐rank test was calculated using the bootstrap method that repeat simulating analyses for re‐extracted datasets (SAS ver.14.0).

## 3 RESULTS

### 3.1 Patient characteristics

Among 465 enrolled patients, 275 patients were with pAF, and 190 with persistent AF. Bigeminal PV potentials were observed in 23 patients before starting ablation procedures (4.9% per patient) (Figure 1B). Clinical characteristics of 23 patients with PV bigeminy and 442 control patients without PV bigeminy are summarized in Table 1. Female and non‐pAF patients were less prevalent in the PV bigeminy group. CHADS_2_ score was significantly less in the PV bigeminy group. In respect to the AF type, PV bigeminy was observed in 18 of 275 in pAF (6.5%) and in 5 of 190 in non‐pAF (2.6%), which is more prevalent in pAF (*P* < .05).

**TABLE 1:** Patient characteristics

	PV bigeminy (+) n = 23	PV bigeminy (‐) n = 442	*P*‐value
Age (y.o.)	59 ± 14	63 ± 12	.22
Female (%)	4 (17.4)	122 (27.6)	.008
non‐pAF (%)	5 (21.7)	180 (40.7)	.012
LAD (mm)	39.0 ± 7.5	41.4 ± 8.0	.18
CHADS2 score	0.5 ± 0.7	1.2 ± 1.1	.023

Abbreviations: LAD: left atrial diameter, PV: pulmonary vein.

### 3.2 Prevalence and localization of PV bigeminy

**FIGURE 2:**
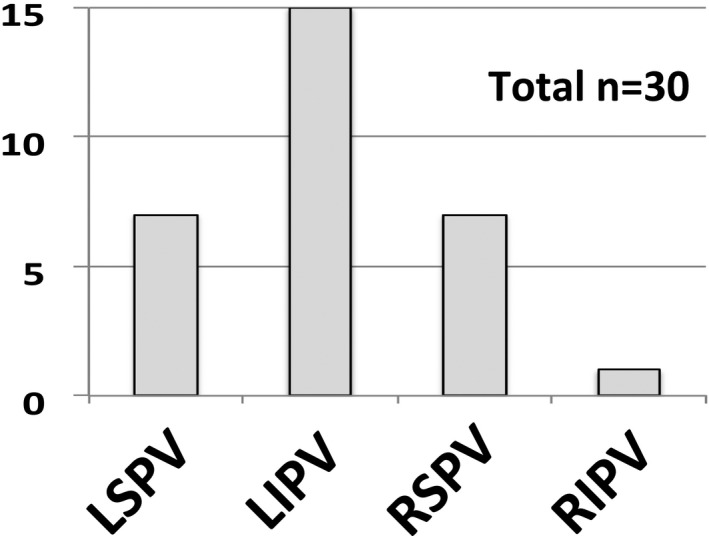
Location of PV bigeminy in 30 PVs of 23 patients

PV bigeminy was observed in 30 individual PVs of 23 patients in total before PVI. PV bigeminy could be spontaneously observed in 14 patients (60.1% of total), whereas it appeared after administration of ISP and/or ATP in the remaining nine patients. The most prevalent of PV bigeminy was left inferior PV (LIPV) (n = 15) followed by left superior PV (LSPV, n = 7), right superior PV (RSPV, n = 7), and right inferior PV (RIPV, n = 1) (Figure 2).

### 3.3 Electrophysiologic property of PV bigeminy

**FIGURE 3:**
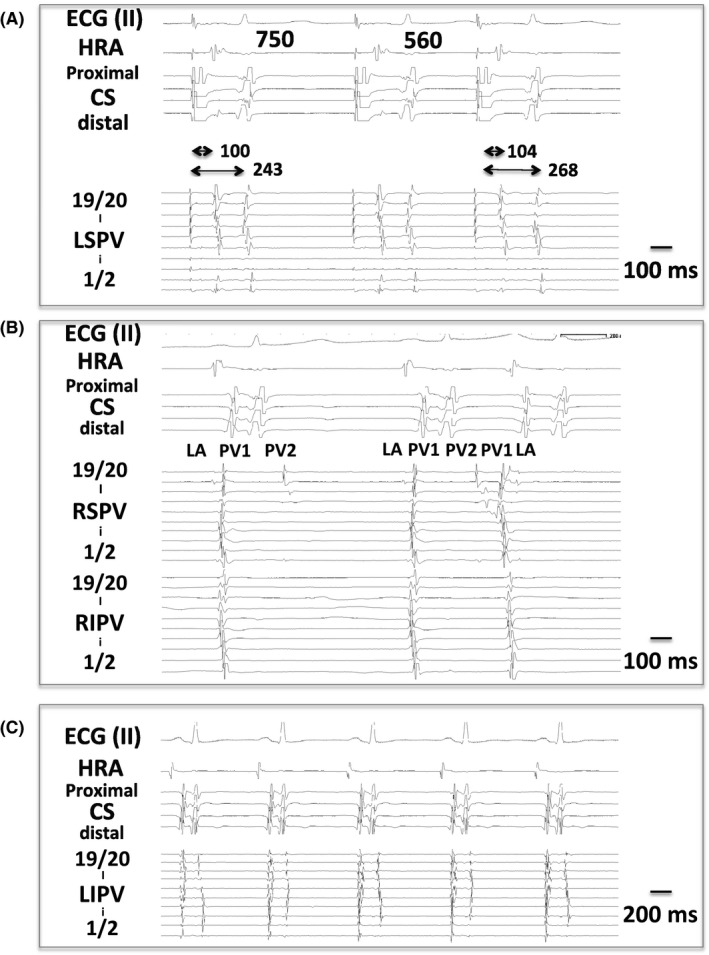
Electrophysiological properties of PV bigeminy. A, Decremental conduction property of PV bigeminy. In this case, effective refractory period of PV2 was 540 ms in response to atrial extra stimulus. B, PV bigeminy with and without following reactivation of the atria, suggesting an establishment of reentry circle via LA‐PV‐PV‐LA. C, Variation in sequence of second PV potentials observed in a patient

**FIGURE 4:**
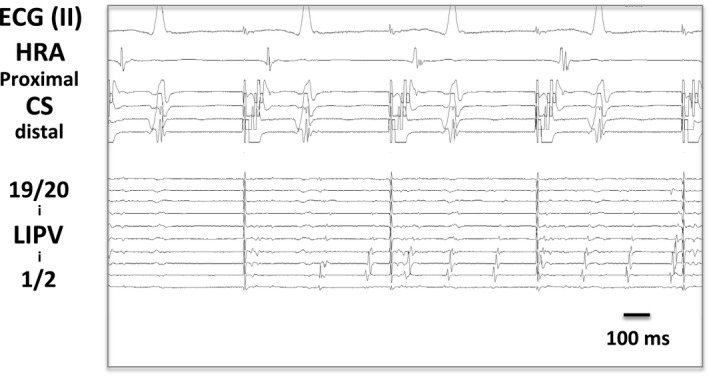
An isolated PV tachycardia in LIPV after PVI in a patient with LIPV bigeminy

Behavior of PV bigeminy in response to a series of atrial extra stimuli could be observed in only five patients since the appearance of PV bigeminy was occasional and sporadic, or easily disappeared under an overdrive atrial pacing in most patients. The first PV potentials (PV1) showed decremental conduction properties as previously described. The second PV potentials (PV2) also showed decremental properties indicated by a prolongation of Spike‐PV2 (S‐PV2) time as well as PV1‐PV2 time in all five patients whose PV bigeminy could follow the overdrive atrial pacing (Figure 3A). Thus, it was suggested that PV bigeminy (PV2) appeared via a reentrant mechanism rather than triggered activity or automaticity. PV2 was followed by an atrial reactivation in some observations (Figure 3B), suggesting an establishment of a reentrant loop between the atrium and the PV during sinus rhythm. In one patient, sequence of PV2 appeared different beat‐by‐beat, suggesting changes of conduction pathway from PV1 to PV2 (Figure 3C). In one patient with LIPV bigeminy, isolated and organized PV tachycardia with a cycle length of 180 ms in the LIPV was observed after PVI, suggesting an establishment of a reentry circuit in the isolated PV (Figure 4).

### 3.4 PV bigeminy and related arrhythmogenic activity

**FIGURE 5:**
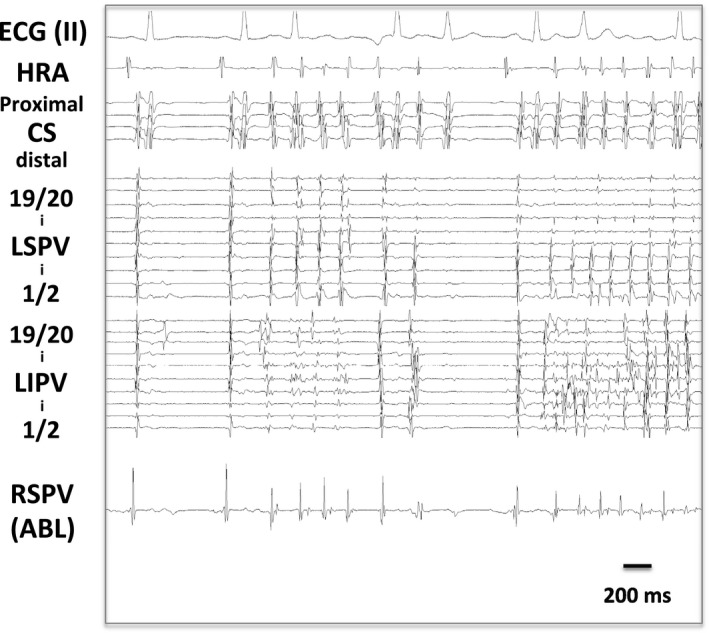
Firing and AF initiation from a PV with bigeminy (LIPV)

**FIGURE 6:**
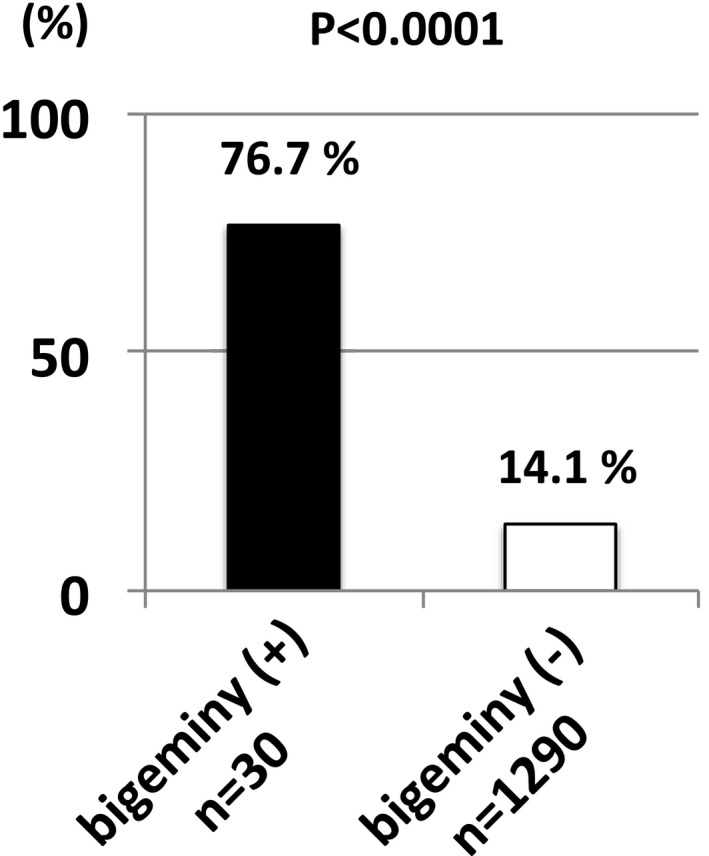
Prevalence of AF initiation from PVs with or without bigeminy

Initiation of AF was observed in 23 PVs of the 30 PVs with PV bigeminy (76.7%), spontaneously or in response to isoproterenol and/or ATP (Figure 5). By contrast, AF initiation was observed in only 182 PVs among the total 1290 PVs (three observed PVs in each) in the control group (14.1%, *P* < .0001 vs PVs with bigeminy, Figure 6). Likewise, in the 23 patients with PV bigeminy, AF initiation from the other PVs without bigeminy was observed only in three. In patient basis, inducibility of AF from any PV could be observed in 22 of 23 patients with PV bigeminy (95.7%), which is significantly more prevalent than that in patients without PV bigeminy (162 patients of 442, 36.7%, *P* < .0001).

### 3.5 Ablation and outcome

All 23 patients with PV bigeminy underwent ablation procedure with simple PVI with no atrial substrate ablation added. PV2 disappeared during the procedure of circumferential PVI, sometimes at the carina region, prior to the completion of PVI with disappearance of PV1 in most of the cases. Four patients among the 23 required multiple sessions because of PV reconnections in 4 and a non‐PV trigger in 1. At 1 year of follow‐up after the final procedures, all patients were free from AF recurrence. Freedom from recurrent AF could be obtained with simple PVI procedure in 22 of 23 patients (95.7%).

## 4 DISCUSSION

The present study characterized PV bigeminy in a series of 23 patients and demonstrated the following findings. First, PV bigeminy was observed in 4.9% of patients undergoing AF ablation and was most prevalently observed in LIPV. Second, electrophysiological behavior of PV bigeminy suggested reentrant mechanisms within or around the PVs at least in part. Finally, PV bigeminy had a strong rationale to the occurrence of AF from the concerned PV.

### 4.1 Prevalence of PV bigeminy

PV bigeminy could be observed in 4.9% in our series of patients, which is not a very rare phenomenon. The patients with PV bigeminy were relatively young and predominantly with pAF and had less comorbidities. Thus, PV bigeminy may not be a consequence of aging or pathological changes induced by cardiac/atrial overload but rather reflect an anatomically provided conduction network within and around the PVs. Hu et al previously reported 22 patients with PV bigeminy of 198 studied patients with pAF (11%),^11^ whereas occurrence of PV bigeminy in the present study was less frequent.

### 4.2 Electrophysiologic property of PV bigeminy

Although the studied number is very small, the pacing study showed that the bigeminal PV potentials exhibited decremental conduction properties in response to atrial extra stimuli, suggesting a sort of reentry rather than triggered activities or ectopic automaticity as responsible mechanisms at least in a certain proportion of cases. This is the first report showing decremental conduction properties of PV bigeminy. PV bigeminy was prevalently seen in the LIPV in our study. In a patient with LIPV bigeminy, an epicardial conduction pathway from PV to epicardial tissue in the CS region was suggested. Taken together, a reentry circuit responsible for PV bigeminy may involve epicardial conduction pathways, such as the ligament of Marshall, as a part of mechanisms.^14^ However, entire mechanisms of PV bigeminy remain unclear and may be different among patients or different between left and right PVs. Indeed, a previous report by Reithman et al did not demonstrate conduction delay of the second PV potential (PV2) in response to overdrive atrial pacing; however, the discrepancy to our results may be due to whether or not performing an extra stimuli pacing protocol.^6^ Higashi et al reported a unique case of PV bigeminy in the LIPV observed during ongoing ablation of left PVI, suggesting a complex and convertible conduction pathway into the PV associated with the appearance of PV bigeminy.^10^ A patient in the present study showed multiple conduction sequences of PV bigeminy, suggesting multiple conduction pathways responsible for PV bigeminy.

### 4.3 Clinical implication

In the present study, PVs with bigeminal activities had extremely high prevalence of firing and AF initiation (76.7%) compared to PVs with no bigeminal activity, suggesting a strong arrhythmogenicity of PVs presenting with bigeminy. In other words, PV bigeminy can be regarded as a strong indicator of AF trigger vein. It is consistent with a previous report by Hu et al^11^ In addition, simple PVI eliminated AF episodes in 22 of 23 patients with PV bigeminy, but one needed non‐PV targeting in the second session. This favorable clinical outcome also strengthened the pathophysiological importance of PVs as AF triggers in patients presenting with PV bigeminy. The favorable clinical outcome in the present study is discrepant form the previous study by Hu et al, which reported that patients with PV bigeminy had a higher AF recurrence rate after catheter ablation compared to those without PV bigeminy.^11^ The reason for this discrepancy is unclear; however, PVI would theoretically be useful for patients with PV bigeminy with an extreme arrhythmogenic activity.

### 4.4 Limitations

There were several limitations in the present study. First, this is a single‐center, observational study and the number of study population was relatively small. The exact prevalence and role of PV bigeminy should be evaluated in a larger number of patients. Second, all four PVs could not be evaluated equally and simultaneously especially during the induction protocol in the present study due to limited number of electrode available in a real‐world clinical setting. Thus, electrical activity and arrhythmogenicity of RIPV may have been underestimated. Indeed, PV bigeminy was observed in RIPV in only one patient. However, PV potentials in all four PVs were at least evaluated in each patient prior to starting ablation procedures. Third, mechanisms of PV potential could not be fully evaluated in this study. The pacing study could not be carried out in the majority of patients because the PV bigeminy is sporadic and not very consistent. In addition, mechanisms of PV bigeminy may be heterogeneous in terms of both anatomical and electrophysiological aspects.

## 5 CONCLUSIONS

PV bigeminy is a unique electrophysiological finding observed in patients with AF suggesting reentrant substrate within the PVs and/or surrounding epicardial tissue. PV bigeminy is a strong indicator of arrhythmogenic vein triggering AF and ensures excellent clinical outcome after PVI.

## CONFLICT OF INTERESTS

The authors declare no conflict of interests for this article.
